# Di-μ-nicotinamide-κ^2^ *N* ^1^:*O*;κ^2^ *O*:*N* ^1^-bis­[aqua­bis­(3-chloro­benzoato-κ^2^ *O*,*O*′)cadmium]

**DOI:** 10.1107/S1600536813015948

**Published:** 2013-06-15

**Authors:** Nihat Bozkurt, Nefise Dilek, Nagihan Çaylak Delibaş, Hacali Necefoğlu, Tuncer Hökelek

**Affiliations:** a Department of Chemistry, Kafkas University, 36100 Kars, Turkey; b Aksaray University, Department of Physics, 68100, Aksaray, Turkey; c Department of Physics, Sakarya University, 54187 Esentepe, Sakarya, Turkey; d Department of Physics, Hacettepe University, 06800 Beytepe, Ankara, Turkey

## Abstract

In the centrosymmetric dinuclear title compound, [Cd_2_(C_7_H_4_ClO_2_)_4_(C_6_H_6_N_2_O)_2_(H_2_O)_2_], the Cd^II^ atom is coord­inated by one N atom from one bridging nicotinamide ligand and one O atom from another symmetry-related bridging nicotinamide ligand, four O atoms from two 3-chloro­benzoate ligands and one water mol­ecule in an irregular geometry. The dihedral angles between the carboxyl­ate groups and the adjacent benzene rings are 6.98 (12) and 2.42 (13)°, while the benzene rings are oriented at a dihedral angle of 4.33 (6)°. Inter­molecular O—H⋯O, N—H⋯O and weak C—H⋯O hydrogen bonds link the mol­ecules into a three-dimensional network. π–π inter­actions, indicated by short centroid–centroid distances [3.892 (1) Å between the pyridine rings and 3.683 (1) Å between the benzene rings] further stabilize the structure.

## Related literature

For niacin, see: Krishnamachari (1974 ▶). For the nicotinic acid derivative *N*,*N*-di­ethyl­nicotinamide, see: Bigoli *et al.* (1972 ▶). For related structures, see: Hökelek *et al.* (2009**a* ▶,b* ▶, 2010*a* ▶,*b* ▶); Necefoğlu *et al.* (2011*a* ▶,*b* ▶); Greenaway *et al.* (1984 ▶).
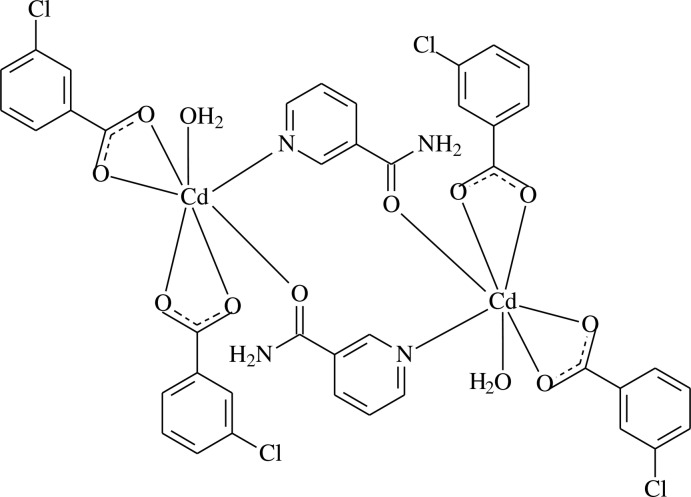


## Experimental

### 

#### Crystal data

[Cd_2_(C_7_H_4_ClO_2_)_4_(C_6_H_6_N_2_O)_2_(H_2_O)_2_]
*M* *_r_* = 1127.32
Triclinic, 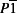

*a* = 7.5835 (2) Å
*b* = 12.3652 (3) Å
*c* = 12.4893 (3) Å
α = 66.878 (2)°
β = 78.678 (3)°
γ = 83.222 (3)°
*V* = 1055.02 (5) Å^3^
*Z* = 1
Mo *K*α radiation
μ = 1.33 mm^−1^
*T* = 296 K
0.38 × 0.24 × 0.12 mm

#### Data collection

Bruker SMART BREEZE CCD diffractometer
Absorption correction: multi-scan (*SADABS*; Bruker, 2012 ▶) *T*_min_ = 0.689, *T*_max_ = 0.853
18369 measured reflections
4310 independent reflections
4106 reflections with *I* > 2σ(*I*)
*R*_int_ = 0.020

#### Refinement

*R*[*F* ^2^ > 2σ(*F* ^2^)] = 0.020
*wR*(*F* ^2^) = 0.052
*S* = 1.09
4310 reflections
296 parameters
103 restraints
H atoms treated by a mixture of independent and constrained refinement
Δρ_max_ = 0.50 e Å^−3^
Δρ_min_ = −0.31 e Å^−3^

### 

Data collection: *APEX2* (Bruker, 2012 ▶); cell refinement: *SAINT* (Bruker, 2012 ▶); data reduction: *SAINT*; program(s) used to solve structure: *SHELXS97* (Sheldrick, 2008 ▶); program(s) used to refine structure: *SHELXL97* (Sheldrick, 2008 ▶); molecular graphics: *ORTEP-3 for Windows* (Farrugia, 2012 ▶); software used to prepare material for publication: *WinGX* (Farrugia, 2012 ▶) and *PLATON* (Spek, 2009 ▶).

## Supplementary Material

Crystal structure: contains datablock(s) I, global. DOI: 10.1107/S1600536813015948/bq2387sup1.cif

Structure factors: contains datablock(s) I. DOI: 10.1107/S1600536813015948/bq2387Isup2.hkl

Additional supplementary materials:  crystallographic information; 3D view; checkCIF report

## Figures and Tables

**Table 1:** Selected bond lengths (Å)

Cd1—O1	2.3234 (14)
Cd1—O2	2.4800 (13)
Cd1—O3	2.5447 (15)
Cd1—O4	2.3110 (16)
Cd1—O5^i^	2.3175 (12)
Cd1—O6	2.3019 (14)
Cd1—N1	2.3384 (14)

Symmetry code: (i) 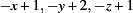.

**Table 2:** Hydrogen-bond geometry (Å, °)

*D*—H⋯*A*	*D*—H	H⋯*A*	*D*⋯*A*	*D*—H⋯*A*
N2—H21⋯O3^i^	0.83 (3)	2.26 (2)	3.026 (2)	155 (2)
N2—H22⋯O2^ii^	0.83 (2)	2.09 (2)	2.913 (2)	170 (2)
O6—H61⋯O1^iii^	0.85 (4)	2.15 (4)	2.897 (2)	146 (3)
O6—H62⋯O3^iii^	0.81 (3)	1.94 (3)	2.710 (2)	158 (3)
C8—H8⋯O5^i^	0.93	2.43	3.158 (2)	135
C10—H10⋯O2^ii^	0.93	2.54	3.403 (3)	154

Symmetry codes: (i) 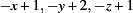; (ii) 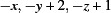; (iii) 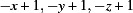.

**Fig. 1.**
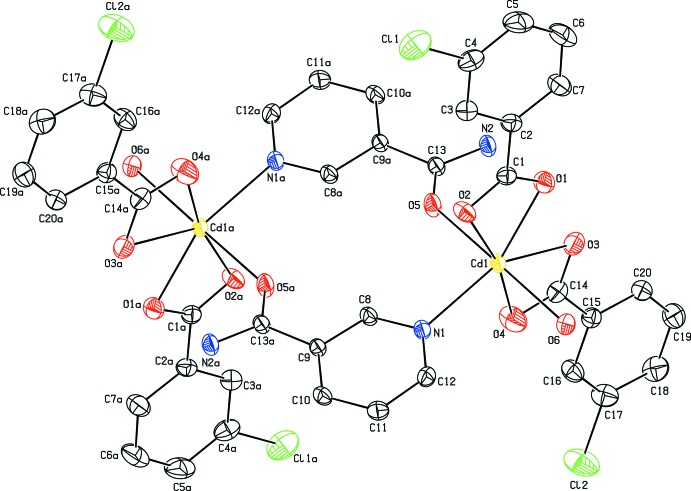
The molecular structure of the title molecule with the atom-numbering scheme. Displacement ellipsoids are drawn at the 50% probability level [symmetry code: (a) 1 - x, - y, 1 - z].

## supplementary crystallographic information

### Comment

As a part of our ongoing investigations of transition metal complexes of nicotinamide (NA), one form of niacin (Krishnamachari, 1974), and/or the nicotinic acid derivative *N*,*N*-diethylnicotinamide (DENA), an important respiratory stimulant (Bigoli *et al.*, 1972), the title compound was synthesized and its crystal structure is reported herein.

The title compound, (I), consists of dimeric units located around a crystallographic symmetry center and made up of two Cd cations, four 3-chlorobenzoate (CB) anions, which act in bidentate modes, two nicotinamide (NA) ligands and two water molecules (Fig. 1). Both of the Cd^II^ centres are seven-coordinated, and the two monomeric units are bridged through the two nicotinamide (NA) ligands about an inversion center. The Cd1···Cd1a [symmetry code: (a) 1 - *x*, - *y*, 1 - *z*] distance is 7.1647 (3)Å. In the molecule, two Cd—O bond distances [2.4800 (13)Å and 2.5447 (15)Å] are significantly longer than the other four, and the average Cd—O bond length is 2.3798 (14)Å (Table 1). The Cd atom is displaced out of the least-squares planes of the carboxylate groups (O1/C1/O2) and (O3/C14/O4) by -0.2003 (1)Å and -0.3909 (1)Å, respectively.

The dihedral angles between the planar carboxylate groups and the adjacent benzene rings *A* (C2—C7) and *C* (C15—C20) are 6.98 (12)° and 2.42 (13)°, respectively, while those between rings *A*, *B* (N1/C8—C12) and *C* are A/B = 80.48 (7)°, A/C = 4.33 (6)°, B/C = 81.80 (7)°.

In (I), the O1-Cd1-O2 and O3-Cd1-O4 angles are 54.22 (4)° and 53.32 (5) °, respectively. The corresponding O—M—O (where M is a metal) angles are 57.75 (2)° in [Cu(C_7_H_5_O_2_F)(C_7_H_4_O_2_F)_2_(C_6_H_6_N_2_O)_2_], (II) (Necefoğlu *et al.*, 2011*a*), 60.32 (4)° in [Co(C_8_H_7_O_3_)_2_(C_6_H_6_N_2_O)(H_2_O)_2_], (III) (Hökelek *et al.*, 2010*a*), 59.02 (8)° in [Zn(C_8_H_8_NO_2_)_2_(C_6_H_6_N_2_O)_2_].H_2_O, (IV) (Hökelek *et al.*, 2009*a*), 60.03 (6)° in [Zn(C_9_H_10_NO_2_)_2_(C_6_H_6_N_2_O)_2_(H_2_O)_2_], (V) (Hökelek *et al.*, 2009*b*), 57.53 (5)°, 56.19 (5)° and 59.04 (4)° in [Zn(C_8_H_7_O_3_)_2_(C_6_H_6_N_2_O)_2_], (VI) (Hökelek *et al.*, 2010*b*), 57.61 (8)° in [Mn_2_(C_7_H_4_O_2_Br)_4_(C_6_H_6_N_2_O)_2_(H_2_O)_2_], (VII) (Necefoğlu *et al.*, 2011*b*) and 55.2 (1)° in [Cu(Asp)_2_(py)_2_] (where Asp is acetylsalicylate and py is pyridine) [(VIII); Greenaway *et al.*, 1984].

In the crystal structure, intermolecular O—H···O, N—H···O and C—H···O hydrogen bonds link the molecules into a three dimensional network (Table 2), in which they may be effective in the stabilization of the structure. The π···π contacts between the pyridine rings and between the benzene rings, Cg2—Cg2^i^ and Cg1—Cg3^ii^ [symmetry codes: (i) 2 - *x*, - *y*, - *z*; (ii) 1 - *x*, 1 - *y*, 1 - *z*, where Cg1, Cg2 and Cg3 are the centroids of the rings *A* (C2—C7), *B* (N1/C8—C12) and *C* (C15—C20), respectively] may further stabilize the structure, with centroid-centroid distances of 3.892 (1)Å and 3.683 (1)Å, respectively.

### Experimental

The title compound was prepared by the reaction of 3CdSO_4_.8H_2_O (1.283 g, 5 mmol) in H_2_O (50 ml) and nicotinamide (1.220 g, 10 mmol) in H_2_O (50 ml) with sodium 3-chlorobenzoate (1.790 g, 10 mmol) in H_2_O (100 ml) at room temperature. The mixture was filtered and set aside to crystallize at ambient temperature for two weeks, giving colorless single crystals.

### Refinement

Atoms H61, H62 (for H_2_O) and H21, H22 (for NH_2_) were located in a difference Fourier map and were freely refined. The C-bound H-atoms were positioned geometrically with C—H = 0.93Å for aromatic H-atoms, and constrained to ride on their parent atoms, with *U*_iso_(H) = *1.2U*_eq_(C).

### Crystal data

**Table:**

[Cd_2_(C_7_H_4_ClO_2_)_4_(C_6_H_6_N_2_O)_2_(H_2_O)_2_]	*Z* = 1
*M**_r_* = 1127.32	*F*(000) = 560
Triclinic, *P*1	*D*_x_ = 1.774 Mg m^−^^3^
Hall symbol: -P 1	Mo *K*α radiation, λ = 0.71073 Å
*a* = 7.5835 (2) Å	Cell parameters from 9868 reflections
*b* = 12.3652 (3) Å	θ = 2.7–28.4°
*c* = 12.4893 (3) Å	µ = 1.33 mm^−^^1^
α = 66.878 (2)°	*T* = 296 K
β = 78.678 (3)°	Block, colorless
γ = 83.222 (3)°	0.38 × 0.24 × 0.12 mm
*V* = 1055.02 (5) Å^3^	

### Data collection

**Table:**

Bruker SMART BREEZE CCD diffractometer	4310 independent reflections
Radiation source: fine-focus sealed tube	4106 reflections with *I* > 2σ(*I*)
Graphite monochromator	*R*_int_ = 0.020
φ and ω scans	θ_max_ = 26.4°, θ_min_ = 1.8°
Absorption correction: multi-scan (*SADABS*; Bruker, 2012)	*h* = −9→9
*T*_min_ = 0.689, *T*_max_ = 0.853	*k* = −15→15
18369 measured reflections	*l* = −15→13

### Refinement

**Table:**

Refinement on *F*^2^	Primary atom site location: structure-invariant direct methods
Least-squares matrix: full	Secondary atom site location: difference Fourier map
*R*[*F*^2^ > 2σ(*F*^2^)] = 0.020	Hydrogen site location: inferred from neighbouring sites
*wR*(*F*^2^) = 0.052	H atoms treated by a mixture of independent and constrained refinement
*S* = 1.09	*w* = 1/[σ^2^(*F*_o_^2^) + (0.0281*P*)^2^ + 0.3773*P*] where *P* = (*F*_o_^2^ + 2*F*_c_^2^)/3
4310 reflections	(Δ/σ)_max_ = 0.002
296 parameters	Δρ_max_ = 0.50 e Å^−^^3^
103 restraints	Δρ_min_ = −0.31 e Å^−^^3^

### Special details

**Table:**

Geometry. All esds (except the esd in the dihedral angle between two l.s. planes) are estimated using the full covariance matrix. The cell esds are taken into account individually in the estimation of esds in distances, angles and torsion angles; correlations between esds in cell parameters are only used when they are defined by crystal symmetry. An approximate (isotropic) treatment of cell esds is used for estimating esds involving l.s. planes.
Refinement. Refinement of F^2^ against ALL reflections. The weighted R-factor wR and goodness of fit S are based on F^2^, conventional R-factors R are based on F, with F set to zero for negative F^2^. The threshold expression of F^2^ > 2sigma(F^2^) is used only for calculating R-factors(gt) etc. and is not relevant to the choice of reflections for refinement. R-factors based on F^2^ are statistically about twice as large as those based on F, and R- factors based on ALL data will be even larger.

### Fractional atomic coordinates and isotropic or equivalent isotropic displacement parameters (Å^2^)

**Table:**

	*x*	*y*	*z*	*U*_iso_*/*U*_eq_	
Cd1	0.460563 (15)	0.713174 (9)	0.502386 (11)	0.02850 (5)	
Cl1	0.20058 (11)	1.03333 (7)	−0.11806 (6)	0.0761 (2)	
Cl2	0.76396 (11)	0.40476 (8)	1.09335 (6)	0.0821 (2)	
O1	0.53731 (18)	0.68689 (12)	0.32444 (12)	0.0428 (3)	
O2	0.31326 (17)	0.81613 (13)	0.32598 (12)	0.0409 (3)	
O3	0.7424 (2)	0.58157 (12)	0.54856 (12)	0.0454 (3)	
O4	0.5701 (2)	0.64166 (16)	0.67878 (14)	0.0635 (5)	
O5	0.35989 (16)	1.12711 (11)	0.55996 (14)	0.0421 (3)	
O6	0.2965 (2)	0.54660 (13)	0.57771 (15)	0.0428 (3)	
H61	0.340 (4)	0.488 (3)	0.631 (3)	0.086 (11)*	
H62	0.266 (4)	0.523 (2)	0.532 (2)	0.059 (8)*	
N1	0.22851 (19)	0.80844 (12)	0.59063 (13)	0.0305 (3)	
N2	0.0768 (2)	1.19067 (14)	0.60728 (15)	0.0359 (3)	
H21	0.110 (3)	1.259 (2)	0.5826 (19)	0.040 (6)*	
H22	−0.033 (3)	1.1805 (19)	0.6266 (19)	0.040 (6)*	
C1	0.4252 (2)	0.76332 (15)	0.27256 (16)	0.0322 (4)	
C2	0.4302 (2)	0.79357 (16)	0.14374 (16)	0.0342 (4)	
C3	0.3235 (3)	0.88734 (17)	0.08028 (17)	0.0386 (4)	
H3	0.2453	0.9300	0.1181	0.046*	
C4	0.3347 (3)	0.91633 (19)	−0.03919 (18)	0.0476 (5)	
C5	0.4493 (4)	0.8553 (3)	−0.0977 (2)	0.0640 (7)	
H5	0.4561	0.8766	−0.1785	0.077*	
C6	0.5542 (4)	0.7616 (3)	−0.0341 (2)	0.0681 (7)	
H6	0.6318	0.7192	−0.0725	0.082*	
C7	0.5445 (3)	0.7307 (2)	0.0856 (2)	0.0497 (5)	
H7	0.6152	0.6672	0.1275	0.060*	
C8	0.2617 (2)	0.91606 (15)	0.57989 (16)	0.0312 (4)	
H8	0.3762	0.9434	0.5445	0.037*	
C9	0.1366 (2)	0.98948 (14)	0.61809 (15)	0.0271 (3)	
C10	−0.0336 (2)	0.94833 (16)	0.67092 (17)	0.0354 (4)	
H10	−0.1225	0.9951	0.6971	0.042*	
C11	−0.0685 (3)	0.83632 (17)	0.68393 (18)	0.0415 (4)	
H11	−0.1813	0.8063	0.7200	0.050*	
C12	0.0650 (2)	0.76927 (15)	0.64305 (17)	0.0351 (4)	
H12	0.0398	0.6939	0.6524	0.042*	
C13	0.1971 (2)	1.10895 (15)	0.59403 (15)	0.0297 (3)	
C14	0.6964 (3)	0.57773 (16)	0.65218 (17)	0.0386 (4)	
C15	0.7945 (2)	0.49355 (16)	0.74759 (17)	0.0346 (4)	
C16	0.7431 (3)	0.48946 (19)	0.86197 (18)	0.0429 (4)	
H16	0.6506	0.5397	0.8790	0.051*	
C17	0.8306 (3)	0.4102 (2)	0.94991 (19)	0.0494 (5)	
C18	0.9655 (3)	0.3336 (2)	0.9270 (2)	0.0538 (5)	
H18	1.0216	0.2793	0.9876	0.065*	
C19	1.0164 (3)	0.3383 (2)	0.8131 (2)	0.0527 (5)	
H19	1.1086	0.2877	0.7965	0.063*	
C20	0.9308 (3)	0.41792 (17)	0.72359 (18)	0.0404 (4)	
H20	0.9652	0.4205	0.6470	0.049*	

### Atomic displacement parameters (Å^2^)

**Table:**

	*U*^11^	*U*^22^	*U*^33^	*U*^12^	*U*^13^	*U*^23^
Cd1	0.02751 (7)	0.02414 (7)	0.03423 (8)	0.00202 (5)	−0.00289 (5)	−0.01339 (5)
Cl1	0.0897 (5)	0.0728 (4)	0.0522 (4)	0.0005 (4)	−0.0339 (3)	−0.0001 (3)
Cl2	0.0835 (5)	0.1212 (6)	0.0423 (3)	0.0194 (4)	−0.0211 (3)	−0.0333 (4)
O1	0.0440 (7)	0.0409 (7)	0.0385 (7)	0.0112 (6)	−0.0088 (6)	−0.0123 (6)
O2	0.0344 (7)	0.0516 (8)	0.0382 (7)	0.0078 (6)	−0.0040 (5)	−0.0224 (6)
O3	0.0644 (9)	0.0350 (7)	0.0377 (8)	−0.0055 (6)	−0.0119 (6)	−0.0122 (6)
O4	0.0594 (10)	0.0753 (11)	0.0474 (9)	0.0313 (9)	−0.0172 (8)	−0.0197 (8)
O5	0.0267 (6)	0.0326 (7)	0.0697 (10)	−0.0027 (5)	0.0032 (6)	−0.0273 (7)
O6	0.0511 (8)	0.0310 (7)	0.0461 (9)	−0.0080 (6)	0.0020 (7)	−0.0172 (7)
N1	0.0301 (7)	0.0272 (7)	0.0351 (8)	0.0009 (5)	−0.0029 (6)	−0.0146 (6)
N2	0.0293 (8)	0.0296 (8)	0.0502 (10)	0.0022 (6)	−0.0013 (7)	−0.0201 (7)
C1	0.0283 (8)	0.0334 (9)	0.0335 (9)	−0.0042 (7)	−0.0019 (7)	−0.0117 (7)
C2	0.0309 (8)	0.0385 (9)	0.0321 (9)	−0.0056 (7)	−0.0004 (7)	−0.0133 (7)
C3	0.0379 (9)	0.0408 (10)	0.0366 (10)	−0.0029 (8)	−0.0055 (8)	−0.0140 (8)
C4	0.0524 (12)	0.0499 (11)	0.0354 (11)	−0.0120 (9)	−0.0107 (9)	−0.0065 (9)
C5	0.0791 (17)	0.0805 (17)	0.0312 (11)	−0.0132 (14)	−0.0016 (11)	−0.0206 (11)
C6	0.0763 (18)	0.0812 (18)	0.0502 (14)	0.0032 (14)	0.0068 (12)	−0.0384 (14)
C7	0.0484 (12)	0.0561 (13)	0.0443 (12)	0.0056 (10)	−0.0013 (9)	−0.0240 (10)
C8	0.0249 (8)	0.0310 (8)	0.0389 (10)	−0.0014 (6)	0.0020 (7)	−0.0176 (7)
C9	0.0259 (8)	0.0270 (8)	0.0295 (8)	0.0012 (6)	−0.0030 (6)	−0.0134 (7)
C10	0.0284 (8)	0.0366 (9)	0.0406 (10)	−0.0002 (7)	0.0045 (7)	−0.0191 (8)
C11	0.0306 (9)	0.0411 (10)	0.0489 (12)	−0.0095 (7)	0.0088 (8)	−0.0178 (9)
C12	0.0357 (9)	0.0282 (8)	0.0403 (10)	−0.0048 (7)	−0.0020 (7)	−0.0127 (7)
C13	0.0273 (8)	0.0300 (8)	0.0342 (9)	0.0005 (6)	−0.0021 (7)	−0.0167 (7)
C14	0.0415 (10)	0.0341 (9)	0.0385 (10)	−0.0038 (8)	−0.0101 (8)	−0.0098 (8)
C15	0.0317 (9)	0.0337 (9)	0.0380 (10)	−0.0029 (7)	−0.0055 (7)	−0.0127 (8)
C16	0.0375 (10)	0.0509 (11)	0.0425 (11)	0.0084 (8)	−0.0089 (8)	−0.0220 (9)
C17	0.0425 (11)	0.0649 (14)	0.0395 (11)	0.0019 (10)	−0.0117 (9)	−0.0173 (10)
C18	0.0424 (11)	0.0559 (13)	0.0525 (13)	0.0086 (9)	−0.0170 (10)	−0.0078 (10)
C19	0.0398 (11)	0.0489 (12)	0.0624 (14)	0.0113 (9)	−0.0064 (10)	−0.0184 (11)
C20	0.0382 (10)	0.0390 (10)	0.0414 (11)	−0.0016 (8)	0.0000 (8)	−0.0155 (8)

### Geometric parameters (Å, º)

**Table:**

Cd1—O1	2.3234 (14)	C3—H3	0.9300
Cd1—O2	2.4800 (13)	C4—C5	1.373 (4)
Cd1—O3	2.5447 (15)	C5—C6	1.383 (4)
Cd1—O4	2.3110 (16)	C5—H5	0.9300
Cd1—O5^i^	2.3175 (12)	C6—H6	0.9300
Cd1—O6	2.3019 (14)	C7—C6	1.379 (3)
Cd1—N1	2.3384 (14)	C7—H7	0.9300
Cd1—C1	2.7496 (18)	C8—H8	0.9300
Cl1—C4	1.739 (2)	C9—C8	1.383 (2)
Cl2—C17	1.740 (2)	C9—C10	1.385 (2)
O1—C1	1.257 (2)	C9—C13	1.497 (2)
O2—C1	1.257 (2)	C10—C11	1.381 (3)
O3—C14	1.256 (2)	C10—H10	0.9300
O4—C14	1.247 (3)	C11—H11	0.9300
O5—Cd1^i^	2.3175 (12)	C12—C11	1.380 (3)
O5—C13	1.241 (2)	C12—H12	0.9300
O6—H61	0.85 (3)	C15—C14	1.499 (3)
O6—H62	0.82 (3)	C15—C16	1.387 (3)
N1—C8	1.333 (2)	C15—C20	1.380 (3)
N1—C12	1.332 (2)	C16—C17	1.377 (3)
N2—C13	1.317 (2)	C16—H16	0.9300
N2—H21	0.83 (2)	C17—C18	1.376 (3)
N2—H22	0.83 (2)	C18—H18	0.9300
C1—C2	1.496 (3)	C19—C18	1.378 (3)
C2—C7	1.386 (3)	C19—H19	0.9300
C3—C2	1.392 (3)	C20—C19	1.382 (3)
C3—C4	1.378 (3)	C20—H20	0.9300
			
O1—Cd1—O2	54.22 (4)	C4—C3—H3	120.3
O1—Cd1—O3	82.45 (4)	C3—C4—Cl1	119.17 (18)
O1—Cd1—N1	137.15 (5)	C5—C4—Cl1	119.11 (18)
O1—Cd1—C1	27.07 (5)	C5—C4—C3	121.7 (2)
O2—Cd1—O3	136.12 (4)	C4—C5—C6	118.8 (2)
O2—Cd1—C1	27.20 (5)	C4—C5—H5	120.6
O3—Cd1—C1	109.10 (5)	C6—C5—H5	120.6
O4—Cd1—O1	135.63 (5)	C5—C6—H6	119.7
O4—Cd1—O2	169.88 (5)	C7—C6—C5	120.5 (2)
O4—Cd1—O3	53.32 (5)	C7—C6—H6	119.7
O4—Cd1—O5^i^	88.27 (6)	C2—C7—H7	119.8
O4—Cd1—N1	87.04 (5)	C6—C7—C2	120.4 (2)
O4—Cd1—C1	162.39 (6)	C6—C7—H7	119.8
O5^i^—Cd1—O1	93.65 (5)	N1—C8—C9	124.08 (15)
O5^i^—Cd1—O2	88.64 (5)	N1—C8—H8	118.0
O5^i^—Cd1—O3	87.51 (4)	C9—C8—H8	118.0
O5^i^—Cd1—N1	90.96 (5)	C8—C9—C10	117.80 (15)
O5^i^—Cd1—C1	90.09 (5)	C8—C9—C13	116.21 (15)
O6—Cd1—O1	89.05 (5)	C10—C9—C13	125.95 (15)
O6—Cd1—O2	96.40 (6)	C9—C10—H10	120.7
O6—Cd1—O3	88.63 (5)	C11—C10—C9	118.53 (16)
O6—Cd1—O4	86.82 (7)	C11—C10—H10	120.7
O6—Cd1—O5^i^	174.96 (6)	C10—C11—H11	120.2
O6—Cd1—N1	89.95 (5)	C12—C11—C10	119.61 (16)
O6—Cd1—C1	94.24 (6)	C12—C11—H11	120.2
N1—Cd1—O2	83.38 (5)	N1—C12—C11	122.44 (16)
N1—Cd1—O3	140.36 (5)	N1—C12—H12	118.8
N1—Cd1—C1	110.52 (5)	C11—C12—H12	118.8
C1—O1—Cd1	95.71 (11)	O5—C13—N2	122.76 (16)
C1—O2—Cd1	88.42 (11)	O5—C13—C9	117.92 (15)
C14—O3—Cd1	86.39 (12)	N2—C13—C9	119.30 (15)
C14—O4—Cd1	97.51 (13)	O3—C14—C15	119.50 (18)
C13—O5—Cd1^i^	136.29 (11)	O4—C14—O3	121.95 (18)
Cd1—O6—H62	118.6 (19)	O4—C14—C15	118.55 (18)
Cd1—O6—H61	115 (2)	C16—C15—C14	119.32 (17)
H62—O6—H61	108 (3)	C20—C15—C14	120.79 (18)
C8—N1—Cd1	115.65 (11)	C20—C15—C16	119.86 (18)
C12—N1—Cd1	126.66 (11)	C15—C16—H16	120.4
C12—N1—C8	117.53 (15)	C17—C16—C15	119.19 (19)
C13—N2—H21	117.5 (16)	C17—C16—H16	120.4
C13—N2—H22	123.5 (15)	C16—C17—Cl2	118.88 (17)
H21—N2—H22	118 (2)	C18—C17—C16	121.3 (2)
O1—C1—O2	121.43 (17)	C18—C17—Cl2	119.75 (17)
O1—C1—C2	118.66 (16)	C17—C18—C19	119.2 (2)
O1—C1—Cd1	57.23 (10)	C17—C18—H18	120.4
O2—C1—Cd1	64.37 (10)	C19—C18—H18	120.4
O2—C1—C2	119.89 (16)	C18—C19—C20	120.2 (2)
C2—C1—Cd1	172.93 (12)	C18—C19—H19	119.9
C3—C2—C1	120.25 (17)	C20—C19—H19	119.9
C7—C2—C1	120.45 (17)	C15—C20—C19	120.2 (2)
C7—C2—C3	119.28 (18)	C15—C20—H20	119.9
C2—C3—H3	120.3	C19—C20—H20	119.9
C4—C3—C2	119.33 (19)		
			
O2—Cd1—O1—C1	2.65 (10)	Cd1—O1—C1—O2	−4.98 (18)
O3—Cd1—O1—C1	−170.02 (11)	Cd1—O1—C1—C2	173.31 (13)
O4—Cd1—O1—C1	−174.29 (11)	Cd1—O2—C1—O1	4.64 (17)
O5^i^—Cd1—O1—C1	−83.03 (11)	Cd1—O2—C1—C2	−173.62 (14)
O6—Cd1—O1—C1	101.24 (11)	Cd1—O3—C14—O4	−8.9 (2)
N1—Cd1—O1—C1	12.33 (14)	Cd1—O3—C14—C15	170.72 (15)
O1—Cd1—O2—C1	−2.64 (10)	Cd1—O4—C14—O3	9.8 (2)
O3—Cd1—O2—C1	7.87 (13)	Cd1—O4—C14—C15	−169.75 (14)
O4—Cd1—O2—C1	165.1 (3)	Cd1^i^—O5—C13—N2	−3.1 (3)
O5^i^—Cd1—O2—C1	92.85 (11)	Cd1^i^—O5—C13—C9	175.50 (12)
O6—Cd1—O2—C1	−86.82 (11)	C12—N1—C8—C9	−1.0 (3)
N1—Cd1—O2—C1	−176.03 (11)	Cd1—N1—C12—C11	−174.35 (15)
O1—Cd1—O3—C14	−171.24 (11)	C8—N1—C12—C11	1.0 (3)
O2—Cd1—O3—C14	−179.82 (10)	O1—C1—C2—C3	−172.19 (17)
O4—Cd1—O3—C14	5.05 (11)	O1—C1—C2—C7	6.0 (3)
O5^i^—Cd1—O3—C14	94.75 (11)	O2—C1—C2—C3	6.1 (3)
O6—Cd1—O3—C14	−82.01 (11)	O2—C1—C2—C7	−175.64 (18)
N1—Cd1—O3—C14	6.26 (14)	C1—C2—C7—C6	−177.4 (2)
C1—Cd1—O3—C14	−176.02 (10)	C3—C2—C7—C6	0.8 (3)
O1—Cd1—O4—C14	0.16 (19)	C4—C3—C2—C1	177.72 (17)
O2—Cd1—O4—C14	−165.6 (3)	C4—C3—C2—C7	−0.5 (3)
O3—Cd1—O4—C14	−5.11 (12)	C2—C3—C4—Cl1	179.81 (15)
O5^i^—Cd1—O4—C14	−93.29 (14)	C2—C3—C4—C5	−0.2 (3)
O6—Cd1—O4—C14	85.54 (14)	Cl1—C4—C5—C6	−179.3 (2)
N1—Cd1—O4—C14	175.66 (14)	C3—C4—C5—C6	0.7 (4)
C1—Cd1—O4—C14	−8.5 (3)	C2—C7—C6—C5	−0.4 (4)
O1—Cd1—N1—C8	−95.81 (14)	C4—C5—C6—C7	−0.4 (4)
O1—Cd1—N1—C12	79.59 (17)	C10—C9—C8—N1	0.1 (3)
O2—Cd1—N1—C8	−87.92 (13)	C13—C9—C8—N1	−177.73 (16)
O2—Cd1—N1—C12	87.48 (15)	C8—C9—C10—C11	0.8 (3)
O3—Cd1—N1—C8	87.85 (14)	C13—C9—C10—C11	178.40 (18)
O3—Cd1—N1—C12	−96.76 (16)	C8—C9—C13—O5	−11.4 (2)
O4—Cd1—N1—C8	88.82 (13)	C8—C9—C13—N2	167.21 (17)
O4—Cd1—N1—C12	−95.78 (16)	C10—C9—C13—O5	170.94 (18)
O5^i^—Cd1—N1—C8	0.60 (13)	C10—C9—C13—N2	−10.4 (3)
O5^i^—Cd1—N1—C12	176.00 (15)	C9—C10—C11—C12	−0.8 (3)
O6—Cd1—N1—C8	175.64 (13)	N1—C12—C11—C10	−0.1 (3)
O6—Cd1—N1—C12	−8.96 (15)	C16—C15—C14—O3	−179.91 (18)
C1—Cd1—N1—C8	−89.86 (13)	C16—C15—C14—O4	−0.3 (3)
C1—Cd1—N1—C12	85.54 (15)	C20—C15—C14—O3	−2.0 (3)
O1—Cd1—C1—O2	175.29 (18)	C20—C15—C14—O4	177.61 (19)
O2—Cd1—C1—O1	−175.29 (18)	C14—C15—C16—C17	178.51 (19)
O3—Cd1—C1—O1	10.47 (12)	C20—C15—C16—C17	0.6 (3)
O3—Cd1—C1—O2	−174.24 (10)	C14—C15—C20—C19	−178.02 (18)
O4—Cd1—C1—O1	13.3 (3)	C16—C15—C20—C19	−0.1 (3)
O4—Cd1—C1—O2	−171.40 (18)	C15—C16—C17—Cl2	−179.44 (16)
O5^i^—Cd1—C1—O1	97.86 (11)	C15—C16—C17—C18	−1.2 (3)
O5^i^—Cd1—C1—O2	−86.85 (11)	Cl2—C17—C18—C19	179.63 (18)
O6—Cd1—C1—O1	−79.54 (11)	C16—C17—C18—C19	1.4 (4)
O6—Cd1—C1—O2	95.75 (11)	C20—C19—C18—C17	−1.0 (4)
N1—Cd1—C1—O1	−171.08 (10)	C15—C20—C19—C18	0.3 (3)
N1—Cd1—C1—O2	4.21 (12)		

Symmetry code: (i) −*x*+1, −*y*+2, −*z*+1.

### Hydrogen-bond geometry (Å, º)

**Table:**

*D*—H···*A*	*D*—H	H···*A*	*D*···*A*	*D*—H···*A*
N2—H21···O3^i^	0.83 (3)	2.26 (2)	3.026 (2)	155 (2)
N2—H22···O2^ii^	0.83 (2)	2.09 (2)	2.913 (2)	170 (2)
O6—H61···O1^iii^	0.85 (4)	2.15 (4)	2.897 (2)	146 (3)
O6—H62···O3^iii^	0.81 (3)	1.94 (3)	2.710 (2)	158 (3)
C8—H8···O5^i^	0.93	2.43	3.158 (2)	135
C10—H10···O2^ii^	0.93	2.54	3.403 (3)	154

Symmetry codes: (i) −*x*+1, −*y*+2, −*z*+1; (ii) −*x*, −*y*+2, −*z*+1; (iii) −*x*+1, −*y*+1, −*z*+1.
